# Hormetic Response to Low-Dose Radiation: Focus on the Immune System and Its Clinical Implications

**DOI:** 10.3390/ijms18020280

**Published:** 2017-01-27

**Authors:** Jiuwei Cui, Guozi Yang, Zhenyu Pan, Yuguang Zhao, Xinyue Liang, Wei Li, Lu Cai

**Affiliations:** 1 Cancer Center, the First Hospital of Jilin University, Changchun 130021, China; ygz@jlu.edu.cn (G.Y.); zhaoyuguang@jlu.edu.cn (Y.Z.); xyliang80@gmail.com (X.L.); liwei66@jlu.edu.cn (W.L.); 2 Department of Radiation-Oncology, the First Hospital of Jilin University, Changchun 130021, China; pzy@jlu.edu.cn; 3 The Pediatric Research Institute, the Departments of Pediatrics, Radiation Oncology, Pharmacology and Toxicology of the University of Louisville, Louisville, KY 40202, USA

**Keywords:** low-dose radiation, hormesis, immune stimulating, immune therapy, autoimmune disease, cancer therapy

## Abstract

The interrelationship between ionizing radiation and the immune system is complex, multifactorial, and dependent on radiation dose/quality and immune cell type. High-dose radiation usually results in immune suppression. On the contrary, low-dose radiation (LDR) modulates a variety of immune responses that have exhibited the properties of immune hormesis. Although the underlying molecular mechanism is not fully understood yet, LDR has been used clinically for the treatment of autoimmune diseases and malignant tumors. These advancements in preclinical and clinical studies suggest that LDR-mediated immune modulation is a well-orchestrated phenomenon with clinical potential. We summarize recent developments in the understanding of LDR-mediated immune modulation, with an emphasis on its potential clinical applications.

## 1. Introduction

Over the past several decades, increasing evidence on the effects of low-dose radiation (LDR) has become available. In contrast to high-dose radiation (HDR), LDR is able to promote growth and development, suppress the aging process, enhance immune functions, and delay cancer progression [1,2]. This interesting phenomenon of the beneficial effects of LDR is often called ‘radiation hormesis’ [3]. The hormetic effect of LDR on the immune system has a great impact on human health, which has attracted the attention of many scientists.

The immune system is one of the most important defenses against environmental insults, and is strongly affected by ionizing radiation. LDR modulates a variety of immune response processes and reveals the properties of immune hormesis. In vitro and in vivo studies have confirmed that the regulatory effect of LDR on innate and adaptive immunity depends on many factors, including the status of immune cells, the microenvironment of the immune system, and the interaction of immune cells [4,5,6]. Preclinical studies have shown LDR to be effective in treating some immune-related diseases [7,8]. For instance, LDR can inhibit the development of infections and malignant tumors by enhancing the immune function of the body [9,10]. On the other hand, LDR can also ameliorate autoimmune diseases, such as arthritis and autoimmune encephalomyelitis, by controlling overactive autoimmune reactions [11,12,13]. These experimental and animal studies suggest that LDR-mediated immune system modulation is a well-orchestrated phenomenon with clinical potential.

Until recently, no consistent evidence has existed with reference to the effects of LDR on the different immune cells. What remains unclear is the circumstances under which certain immune cell types are most sensitive to LDR, and how LDR-induced effects on different immune cells can potentially be used in the prevention and therapy of immune-related diseases. Thus, it is worthwhile to further clarify and provide a prospective overview of the potential applications of LDR in immune-related diseases. We review recent developments in the understanding of LDR immune modulation, with emphasis on its potential clinical applications.

## 2. The Hormetic Effect of LDR on the Immune System

The human immune system mainly includes innate immunity and adaptive immunity. The innate immune system is the first line of defense for the body, taking immediate action in response to invading pathogens. This system primarily involves natural killer (NK) cells, macrophages, and dendritic cells (DCs). A more evolved adaptive immunity creates immunological memory after an initial response to a specific pathogen and then leads to an enhanced response to that pathogen. This process involves cellular and humoral immune cells (T cells and B cells). It has been demonstrated that LDR enhances the immune response by augmenting the proliferation-reactive response of immune cells to mitogenic stimulation, altering immune cell populations and cytokine release as well as enhancing the interaction of innate and adaptive immune cells [5,14,15,16,17].

### 2.1. The Hormetic Effect of LDR on Innate Immunity

Cells of the innate immune system act as the first line of defense against invading pathogens. The hormetic effect of LDR on innate immunity was mainly reported as the modulation of innate immune cells by LDR. 

#### 2.1.1. The Effect of LDR on NK Cells

As innate immune effectors, NK cells play a key role in immune surveillance against viral, bacterial, fungal, and protozoan infections [18]. Through the secretion of pro-inflammatory cytokines and cytotoxic activity, NK cells can eliminate infected or transformed cells. Our and other studies in vitro and in vivo indicate that LDR may enhance the activity of NK cells by stimulating cell proliferation and promoting the cytotoxic function of NK cells [19,20,21]. In addition, Sonn et al. demonstrated that LDR was capable of synergizing NK cytotoxicity indirectly among NK cells previously exposed to cytokines, such as low-level interleukin-2 (IL-2) or foreign pathogens [22]. LDR could also influence NK cell-mediated cytotoxicity indirectly by stimulating the endocrine system and the central nervous system [23].

Despite many reports of LDR-induced activation of NK cells, the molecular mechanisms driving this phenomenon remain obscure and controversial. Sonn et al. reported that enhancement of NK cytotoxicity induced by LDR was not due to changes in the rate of early or late apoptosis of NK cells or alterations in NK-activating receptors (NK1.1, NKG2D, CD69, and 2B4) [22]. However, another study demonstrated that LDR decreased apoptosis in NK cells [24]. Additionally, the possible mechanism of increased activity of NK cells induced by LDR was associated with the elevation of glutathione production and secretion of cytokines, such as IL-2, IL-12, interferon-γ (IFN-γ), and tumor necrosis factor-α (TNF-α) [14,25]. A study by our group found that LDR-induced NK cell activation was associated with the p38/MAPK (mitogen-activated protein kinases) signaling pathway [21]. Further studies of the precise mechanisms underlying LDR-induced NK cell activation are needed to apply this effect to treating immune-related diseases.

#### 2.1.2. The Effect of LDR on Macrophages

Macrophages are traditional innate immune cells that play critical roles in the clearance of pathogens and the maintenance of tissue homeostasis [26,27,28]. Specific tissue and micro-environmental signals trigger differentiation and specialized activation of macrophages into either “classical” (M1) or “alternative” (M2) activated phenotypes. M1 macrophages can activate T helper type 1 (Th1) cells to enhance the immune response [29], while M2 macrophages mediate an anti-inflammatory response via type 2 helper cells (Th2) to facilitate tissue remodeling processes like angiogenesis [30]. Importantly, they also promote tumor cell growth, angiogenesis, invasion, and metastasis [31,32,33], known as tumor-associated macrophages (TAMs) [34].

It has been described that LDR programs the differentiation of inducible nitric oxide synthase (iNOS^+^) M1 macrophages that orchestrate cytotoxic T cell recruitment into and killing within solid tumors [9,12,35]. In fact, LDR also has an effect on the transformation of different macrophage cell types. Prakash et al. found that LDR could promote TAM differentiation to the M1 phenotype; this differentiation was characterized by induction of M1-associated effecter cytokines as well as a reduction in pro-tumorigenic and M2-associated effecter cytokines [35]. On the contrary, LDR could induce M1 phenotype to M2 macrophage [36]. Mechanically, the LDR-induced differentiation of macrophages was partly associated with the induction of endothelial activation and the expression of Th1 chemokines, as well as the suppression of the production of angiogenic, immunosuppressive, and tumor growth factors [35]. Inhibition of the iNOS pathway and nitric oxide (NO) production, reduction of oxidative burst activity and superoxide production, and inhibition of protein kinase-B (AKT) and p38/MAPK phosphorylation were also involved in the effect of LDR on macrophage function [37,38,39,40]. These in vivo and in vitro findings strongly suggest an influence of LDR on macrophage polarization and function. 

#### 2.1.3. The Effect of LDR on Dendritic Cells

Dendritic cells (DCs) are the most effective and professional antigen-presenting cells (APC) in the innate immune system, which initiates the adaptive immune response [41]. The immunological activity of DCs depends on DC differentiation and maturation status. Because of the special and complex roles of DCs in the immune system, reports on the effects of LDR on DCs are conflicting. A study conducted by Jahns et al. first analyzed the direct effect of LDR on human DCs [42]. They showed that irradiation of DC precursors with 0.5 Gy in vitro does not influence the surface marker expression or cytokine profile of immature DCs or of mature DCs after lipopolysaccharide treatment [42]. In contrast, Shigematsu et al. reported that the 0.05 Gy-pre-irradiated DCs exhibited the highest proliferation capacity of T cells, and augmented the production of IL-2, IL-12, and IFN-γ [43]. Similarly, these authors found that LDR did not augment the expression of major histocompatibility complex (MHCs) or costimulatory molecules on DCs, such as cluster of differentiation 1a (CD1a), CD40, CD80, CD86, and intracellular adhesion molecules (ICAMs) [43]. The mechanism behind this result remains to be determined.

So far, it indicates that LDR may stimulate innate immunity cells, which further activate the cells of adaptive immunity, leading to the enhancement of the immune response. However, in light of the inconsistencies in present reports, which may be caused by inter-laboratory variations in irradiation dose, dose rate, and irradiation time, further studies are required to confirm the effects and mechanisms of LDR on DCs.

### 2.2. The Hormetic Effect of LDR on Adaptive Immunity

Alongside the innate immune system, adaptive immunity is a major system that creates immunological memory after an initial response to a specific pathogen. This memory leads to an enhanced response to subsequent encounters with the pathogen. The adaptive immunity includes both cell-mediated immunity components and humoral immunity components, which mainly involve T cells and B cells, respectively. 

#### 2.2.1. The Effect of LDR on T Cells

The T cell plays a central role in cell-mediated immunity. There are three broad categories of T cells. In vitro and in vivo experiments confirmed that LDR could increase the subpopulations and enhance the response of CD4+ T cells [44,45,46,47]. Similarly, an enhanced CD8+ cytotoxic T cells (CTLs) response following LDR has also been observed [17,48]. The molecular mechanisms underlying the regulatory effect of LDR on T cell immunity may involve the observations of activated survival/signaling proteins (e.g., nuclear factor-κB (NF-κB), p38/MAPK, and c-Jun N-terminal kinase (JNK)), and an increased capacity of T cells to produce immune enhancing cytokines (IL-2 and IL-4) while decreasing production of a major immunosuppressive cytokine (transforming growth factor-β1—TGF-β1) [49]. LDR-induced changes in these signaling networks constitute a unique pattern responsible for the enhanced T cell immunity, demonstrated by increasing the expression levels of several CD markers and chemokines [50]. For instance, the reported CD makers that are upregulated at the protein expression level by LDR include T cell receptor (TCR)/CD3 [51], CD2 [52], CD4 [6], and CD28 [53]. In addition, LDR is able to increase the expression of APC and T cell surface markers, which results in a reduction of self-tolerance induced by tumor cells and induction of anti-tumor immunity.

Some studies also show that the number and function of regulatory T cells (Tregs) were markedly decreased in mice or rats following treatment with LDR [54], which eventually enhanced the antitumor immunity [55]. The mechanism of LDR-induced effects on Tregs is not well explored yet. Wang et al. showed that the cell surface expression of cytotoxic T lymphocyte-associated antigen-4 (CTLA-4) moderately decreased on Tregs in mice [55]. On the other hand, the best studied cytokine, IL-10, which is the most relevant molecule mediating Treg suppressor activity, was also downregulated by LDR [56,57]. These phenomena may be the main drivers of LDR-induced enhancement of immune response through Tregs.

On the contrary, in an animal model of autoimmune disease, it was observed that LDR could stimulate a selective retention/expansion of Tregs capable of producing immunosuppressive activity to control autoimmune disease [58]. Obviously, the effects of LDR on Tregs are opposite in these two types of diseases, which may result from differing microenvironments of the diseases, or differences in LDR dosage and exposure style.

#### 2.2.2. The Effect of LDR on B Cells

B cells are the major cells involved in the production of antibodies that circulate in blood plasma and lymph; this process is known as humoral immunity. Reportedly, LDR can affect many aspects of B cell behavior. At the same time, the molecular mechanisms underlying the effect of LDR on B cells have also been explored. 

LDR could enhance B lymphoblast proliferation by elevation of cyclin E and cyclin-dependent kinase 2 (CDK2), as well as elevation of the phosphorylation level of Ikaros protein, a member of the zinc finger-containing transcription factor family [59]. LDR can also modulate B cell differentiation through the activation of NF-κB and the induction of cell differentiation molecule CD23 expression [60]. In addition, several reports state that LDR increased global genomic DNA methylation, induced release of extracellular ataxia telangiectasia mutated kinase (ATM), and promoted a metabolic shift from oxidative phosphorylation to aerobic glycolysis, which led to increased radiation resistance in human B cells [34,61,62]. These findings indicate that LDR has the potential to enhance B cell immune response when it is used prior to conventional radiotherapy.

Above all, LDR-induced immune hormesis occurs mainly via alterations in innate and adaptive immune cells [63]. These effects are probably the reason, at least in part, for the decreased incidence of certain cancers or an extended life span in some people exposed to LDR in the area with naturally high background radiation or an occupational environments with increased levels of radiation. However, a few studies showed conflicting results [64,65]. These inconsistencies suggest that sophisticated models are required to accurately predict the health consequences of occupational, environmental, and clinical exposure.

## 3. Clinical Implications of an LDR Effect on the Immune System

Many human diseases have causes related to immune dysfunction. The immune function decline occurs when the immune system is not as strong as normal, resulting in malignant tumors or life-threatening infections. On the contrary, autoimmune disease results from a hyperactive immune system attacking normal tissue. At present, the therapeutic drugs for these immune-related diseases are limited. Most of these drugs are designed to regulate one of the multiple steps in the process of the immune response, thereby leading to other adverse reactions associated with immune disorders.

In contrast with these traditional therapeutic drugs, the regulatory effect of LDR on the immune system depends on the immune microenvironment of the body. In different immune-related diseases, the integrated regulation of LDR on the immune system enables the immune system to achieve balance, thereby treating the disease. In recent decades, researchers have systematically investigated the effects of LDR on animal models of these diseases; these efforts are contributing to a theoretical basis for the clinical application of low-dose radiotherapy.

### 3.1. The Application of LDR on Autoimmune Diseases

In recent years, a large number of studies in vitro and in vivo have demonstrated that LDR has the potential to be used in the clinical treatment of autoimmune diseases. For instance, it has been observed that repeated LDR significantly inhibited osteoclastic activity and subsequent bone resorption in patients with rheumatoid arthritis [66,67,68]. In mice with autoimmune encephalomyelitis or asthma, LDR treatment improved symptoms and suppressed disease development [13,69]. While the cancer risk posed by LDR remains controversial, progress in understanding LDR benefits to immune regulation may help establish it as an alternative treatment for autoimmune diseases.

Since inflammation is an important pathophysiological process of autoimmune disease, the anti-inflammatory effect of LDR plays a key role in the mechanism by which LDR treats autoimmune disease [70,71,72]. LDR could inhibit the expression of proinflammatory cytokines, upregulate the proportion of Tregs, and reduce the production of autoantibodies to achieve the effect of anti-inflammatory response [11,13,67,71,73,74,75], thereby controlling the development of autoimmune disease.

However, LDR could also reduce the Treg population, which is associated with anti-tumor immunity, in mice with tumors [55,76]. A difference may exist between LDR-induced effects in mice with tumors and autoimmune disease mouse models. In addition, when the pathogenic factor of autoimmune disorder is external antigen, LDR may enhance, not reduce, auto-antibody production, which could exacerbate the disease pathology [77]. 

Overall, LDR seems to exert diverse hormetic effects on immune cells. Since the human immune system is complex, it remains unclear which LDR effects act on immune cells for particular autoimmune diseases. Further experimental studies on the molecular mechanisms underlying the effects of LDR on the immune system are necessary for effectively using LDR as an autoimmune disease treatment. In addition, based on the current linear no-threshold (LNT) theory for the risk of radiation carcinogenesis that is extrapolated from observations of effects at high or moderate doses, there is concern about whether LDR can increase cancer risk, which significantly impedes the further validation and acceptance of LDR for benign autoimmune illnesses However, most epidemiology studies performed on nuclear plant workers exposed to LDR do not have adequate statistical power regarding an increased risk of cancer [78,79]. Therefore, we believe that LDR will be an optimal radio-therapeutic treatment regimen for patients with benign autoimmune illnesses.

### 3.2. The Application of LDR on Malignant Tumors

The development and establishment of tumor cells in the body can suppress the immune system, while activation of both innate and adaptive immunity has been shown to boost anticancer activity. Conventional radiotherapy and chemotherapy are well-established and effective forms of cancer treatment. However, the success of radiotherapy and chemotherapy is limited by systemic and normal tissue toxicity, including the inhibition of immune function. Therefore, upregulation of immune surveillance by LDR offers an anticancer therapeutic strategy.

Epidemiological and experimental results indicate that LDR can inhibit tumor growth, metastasis, and occurrence by enhancing the immune response [80]. For example, in a well-recognized model of thymic lymphoma induced by HDR, an LDR treatment (0.05 to 0.2 Gy) 6–24 h preceding each fractionated HDR could significantly reduce the lymphoma incidence; this effect was shown to be accompanied by enhanced anticancer immunity [81]. Low-dose X-rays and γ-rays in different strains of mice decrease rates of tumor growth and metastasis, which correlates well with immune enhancement [38,39,82]. In addition to experimental animal studies, epidemiological surveys of humans showed that inhabitants of locales with high background radiation have lower cancer mortality than residents of areas with a normal background radiation environment, which was also found to correlate with immune enhancement [83,84,85,86]. Several recent investigations found that LDR was more effective in cancer treatment than conventional radiotherapy, because of the fact that LDR could stimulate the immune system, in contrast to high radiation doses, which usually suppress it.

These results support the use of LDR as a regimen for cancer prevention and clinical treatment. Nonetheless, the issue of whether LDR could also induce the same hormesis in cancer cells warrants discussion. This information will be important since exposure times and doses of LDR can be manipulated to favor anti-cancer effects. Our group has demonstrated, for the first time, that LDR-induced proliferative effects are absent in cancer cells, including leukemia and solid tumor cells, in vitro and in vivo [87]. The mechanism of this absence was associated with differential LDR activation of the MAPK/ERK (extracellular signal regulated kinase), phosphatidylinositol 3-kinase (PI3K)/AKT, and ATM signaling pathways [88,89]. 

In addition, other studies demonstrate that the lack of hormesis induced by LDR has been observed in cancer cells [90,91,92]. However, there are a few studies that had contrary findings: tumors in LDR-irradiated rats grew faster than those in non-irradiated rats [93]; LDR-induced hormesis was also observed in two breast carcinoma cell lines from 2 to 24 h post-irradiation [94]. Apparently it is not a common phenomenon that LDR was unable to induce hormesis in cancer cells. These controversial findings suggest the urgency for us to further explore whether the dose level and dose rates of LDR favoring the induction of hormesis in normal immune cells are different from those in tumor immune responses; whether the duration of hormesis induced by LDR is different between tumor and normal immune cells; and whether all these variables are also different among tumors. Therefore, to further understand the underlying molecular mechanisms for these differences is urgent since these will be the theoretic basis for the clinical application of LDR in anti-cancer therapy. 

## 4. Conclusions and Perspectives

**Figure 1:**
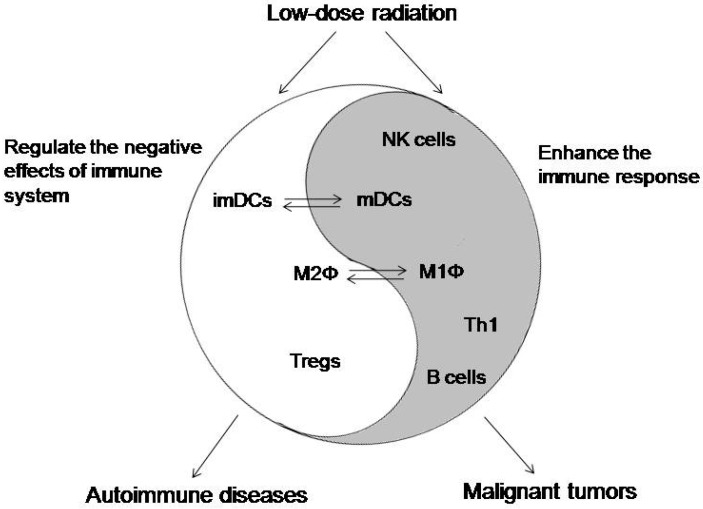
A model of LDR-induced hormetic effects on the immune system and their clinical indication. LDR has been shown to have dual effects on the regulation of immune hormesis. On the one hand, LDR could enhance the cytotoxicity of natural killer (NK) cells, promote the differentiation of mature dendritic cells (mDCs) and M1 macrophages (M1Φ), and activate T helper type 1 (Th1) cells and B cells (gray part of the figure), leading to the enhancement of the immune response, which can be applied in the treatment of malignant tumors. On the other hand, LDR regulates negative effects of the immune response by inhibiting the transformation of immature DCs (imDCs) to mDCs, inducing the differentiation of M2Φ, and stimulating a retention/expansion of Tregs (white part of the figure). This immunosuppressive activity can be used to control autoimmune diseases.

The literature increasingly indicates that LDR modulates a variety of immune responses, including innate and adaptive immunity. At the same time, LDR has shown dual effects in the regulation of immune hormesis (Figure 1), depending on the microenvironment of the immune system and the interaction of immune cells; the underlying definite molecular mechanisms remain ambiguous. The preclinical and clinical studies of the specific effects of LDR on the immune system indicate potential efficacy in the treatment of some immune-related diseases, especially autoimmune diseases and malignant tumors.

It should be mentioned that cancer and autoimmune diseases are fundamentally different. In autoimmune diseases, the immune response hyperactivates against a self-antigen, leading to tissue injury, while in cancer it is suppressed and unable to eradicate the transformed self-cells. Therefore we have to further explore the effects of LDR on the immune system. Using the proper dose of ionizing radiation can be part of immunotherapy in future treatment of both benign autoimmune diseases and malignant tumors. Compared with the traditional treatment of these two types of diseases, low-dose radiotherapy is simple, convenient, and easy to implement for most medical institutions.

However, unresolved questions, e.g., the optimal dose and timing of LDR therapy for different diseases, motivate further investigation. In addition, the pathogenesis of various diseases is complex and diverse. The effect of LDR on immune cells in vitro will be different from that on the body’s immune system. The therapeutic effects of LDR on a disease will be affected by both the disease itself and the microenvironment of the body.

Therefore, to maximize the effectiveness of the immune response to LDR we need additional clinical trials with appropriate design and statistical analysis. To establish optimal protocols we need to maximize the magnitude and duration of immune response along with minimal marrow radiation via reducing irradiated body volume. We also need proper clinical trials to define the effectiveness of LDR immune stimulation in patients who have other disease-related immune responses.
